# Differences in management approaches for lupus nephritis within the UK

**DOI:** 10.1093/rap/rkae017

**Published:** 2024-02-09

**Authors:** Sara T Ibrahim, Christopher J Edwards, Michael R Ehrenstein, Bridget Griffiths, Caroline Gordon, Peter Hewins, David Jayne, Liz Lightstone, Zoe McLaren, Benjamin Rhodes, Edward M Vital, John A Reynolds

**Affiliations:** Rheumatology Research Group, Institute of Inflammation and Ageing, University of Birmingham, Birmingham, UK; Internal Medicine and Nephrology Department, Faculty of Medicine, Alexandria University, Alexandria, Egypt; NIHR Southampton Clinical Research Facility, University Hospital Southampton, Southampton, UK; Department of Rheumatology, University College London, London, UK; Department of Rheumatology, Freeman Hospital, Newcastle upon Tyne Hospitals NHS Foundation Trust, Newcastle upon Tyne, UK; Rheumatology Research Group, Institute of Inflammation and Ageing, University of Birmingham, Birmingham, UK; Department of Rheumatology, Sandwell and West Birmingham NHS Trust, Birmingham, UK; Department of Renal Medicine, Queen Elizabeth Hospital, University Hospitals Birmingham NHS Foundation Trust, Birmingham, UK; Department of Medicine, University of Cambridge, Cambridge, UK; Centre for Inflammatory Disease, Department of Immunology and Inflammation, Imperial College London, London, UK; Department of Rheumatology, Liverpool University Hospitals NHS Foundation Trust, Liverpool, UK; Rheumatology Department, University Hospitals Birmingham NHS Foundation Trust, Birmingham, UK; Leeds Institute of Rheumatic and Musculoskeletal Medicine, University of Leeds, Leeds, UK; NIHR Leeds Biomedical Research Centre, Leeds Teaching Hospitals NHS Trust, Leeds, UK; Rheumatology Research Group, Institute of Inflammation and Ageing, University of Birmingham, Birmingham, UK; Department of Rheumatology, Sandwell and West Birmingham NHS Trust, Birmingham, UK

**Keywords:** lupus nephritis, treatment, UK, refractory

## Abstract

**Objectives:**

Outcomes of therapy for LN are often suboptimal. Guidelines offer varied options for treatment of LN and treatment strategies may differ between clinicians and regions. We aimed to assess variations in the usual practice of UK physicians who treat LN.

**Methods:**

We conducted an online survey of simulated LN cases for UK rheumatologists and nephrologists to identify treatment preferences for class IV and class V LN.

**Results:**

Of 77 respondents, 48 (62.3%) were rheumatologists and 29 (37.7%) were nephrologists. A total of 37 (48.0%) reported having a joint clinic between nephrologists and rheumatologists, 54 (70.0%) reported having a multidisciplinary team meeting for LN and 26 (33.7%) reported having a specialized lupus nurse. Of the respondents, 58 (75%) reported arranging a renal biopsy before starting the treatment. A total of 20 (69%) of the nephrologists, but only 13 (27%) rheumatologists, reported having a formal departmental protocol for treating patients with LN (*P* < 0.001). The first-choice treatment of class IV LN in pre-menopausal patients was MMF [41 (53.2%)], followed by CYC [15 (19.6%)], rituximab [RTX; 12 (12.5%)] or a combination of immunosuppressive drugs [9 (11.7%)] with differences between nephrologists’ and rheumatologists’ choices (*P* = 0.026). For class V LN, MMF was the preferred initial treatment, irrespective of whether proteinuria was in the nephrotic range or not. RTX was the preferred second-line therapy for non-responders.

**Conclusion:**

There was variation in the use of protocols, specialist clinic service provision, biopsies and primary and secondary treatment choices for LN reported by nephrologists and rheumatologists in the UK.

Key messages

- Preferred treatment for lupus nephritis in the UK varies among rheumatologists and nephrologists.
- Hydroxychloroquine use was lower than expected and few respondents reported the use of combination therapies.
- Access to local protocols, multidisciplinary team meetings and specialized nurses needs to be improved.

## Introduction

SLE is an autoimmune disease with variable prevalence in different geographical regions and demographic subgroups [1]. In the UK, the estimated prevalence is ≈97/100 000 in the adult population and differs between regions, principally due to differences in ancestral distributions [2]. Up to 60% of SLE patients will develop LN in their disease course [3, 4]. In 2001, the prevalence of biopsy-proven LN in the general population of northwest England was 4.4/100 000 [5]. Appropriate management of LN is essential, as patients with LN have a higher risk of death than SLE patients without nephritis and inadequately treated patients are more likely to develop end-stage renal disease (ESRD), which is associated with high healthcare costs and reduced health-related quality of life [3].

Although there are several guidelines for the treatment of patients with LN, including those from the British Society for Rheumatology (BSR) [6], EULAR, European Renal Association–European Dialysis and Transplant Association (ERA-EDTA) [7] and Kidney Disease: Improving Global Outcomes (KDIGO) group [8], the management of LN is still considered a challenge. Physicians must take into consideration multiple factors such as age and reproductive status of the patient, clinical presentation and histopathological findings, as well as both prior treatment and patients’ preferences before deciding the appropriate management for their patients. Furthermore, patients may show different responses to different treatment protocols and the factors responsible for this are still not fully understood [9].

Previous randomized controlled trials (RCTs) demonstrate similar outcomes for MMF and CYC [10, 11] in remission induction, and MMF is commonly regarded as the standard of care for most LN patients. While in a recent trial, MMF and prednisolone alone demonstrated that only 23% of patients achieved the 12-month treatment [12], there may be increased efficacy in combining MMF with newer agents [13, 14]. Although previous RCTs found no benefit in combining MMF with either rituximab (RTX) or abatacept [15, 16], two recent studies showed additional benefit in adding belimumab to a background of MMF, or CYC, and prednisolone [13] or adding voclosporin to MMF and prednisolone [12].

As several treatment options are available, there is a need to understand whether there is variation in preferred treatment approaches. In addition to the choice of immunosuppressive agent, outcomes in LN may also be influenced by the provision of specialist services such as combined clinics and multidisciplinary team (MDT) meetings. We therefore aimed to determine variations in the management of LN and access to specialist services by rheumatologists and nephrologists in the UK in 2022.

## Methods

We conducted an online survey of simulated LN case scenarios for rheumatologists and nephrologists within the UK. The survey comprised 23 questions, including some questions to gather information about local services in the hospitals where physicians treat LN, such as the presence of specialized lupus nurses, departmental protocols for LN and combined rheumatology/nephrology clinics. Most of the questions were developed to identify the treatment preferences of the physicians for class IV and class V LN. For class IV, the scenarios covered treatment of pre-menopausal *vs* post-menopausal patients, patients with high activity in renal biopsy *vs* patients with high chronicity, and patients with renal impairment *vs* patients without renal impairment. For class V, the scenarios covered patients with nephrotic-range proteinuria *vs* mild proteinuria (with mild renal impairment in both cases). For both class IV and class V LN, both the first-choice treatment and the second-line treatment for non-responders were ascertained. For more details about the survey questions and the clinical scenarios, see Supplementary Data S1, available at *Rheumatology Advances in Practice* online. The survey content was developed and validated by a BILAG working group of rheumatologists and nephrologists. The survey was created using Jisc Online Surveys version 2.0 (Jisc, Bristol, UK) and circulated via local and regional networks and online through the UK Kidney Association and BSR bulletins. Participants gave implied consent by responding to the survey. The survey was open for 7 weeks (from 28 June 2022 to 17 August 2022) and respondents’ answers were extracted and analysed using SPSS version 23 (IBM, Armonk, NY, USA), licensed to Alexandria University. Sankey diagrams were created using SankeyMATIC. Descriptive statistics were used with the chi-squared test, with Monte Carlo simulation to compare between groups. This provides an unbiased estimate of the true *P*-value where an asymptotic method may be less accurate.

The protocol was reviewed by the University of Birmingham’s ethical committee (Application for Ethical Review: ERN_2022-0188), who deemed that no further ethical review was required.

## Results

We received 94 responses, but of these, 17 participants did not treat patients with LN and therefore did not complete the survey. Of the 77 remaining respondents, 67 (87%) were consultants (38 were rheumatologists and 29 were nephrologists) and 10 (13%) were rheumatology trainees. No nephrology trainees completed the survey. In this context, trainees are physicians who are in higher specialist training but who are not yet able to practice independently.

In terms of services to support the treatment of LN, 33 (42.8%) reported the presence of a departmental protocol for treating LN, 37 (48.0%) reported the presence of a joint clinic between nephrologists and rheumatologists, 54 (70.0%) reported the presence of an MDT to discuss cases of LN and 26 (33.7%) reported the presence of a specialized lupus nurse in their workplace.

The numbers of new LN patients managed by each respondent over the past 12 months were <5 patients [46 (59.7%)], 5–10 patients [13 (16.8%)], 11–20 patients [10 (12.9%)], 21–30 patients [3 (3.9%)] and >30 patients [5 (6.5%)]. A total of 58 (75%) respondents reported arranging a renal biopsy before starting treatment, even if the diagnosis was clearly LN.

### Treatment choices for class IV LN

**Figure 1.**
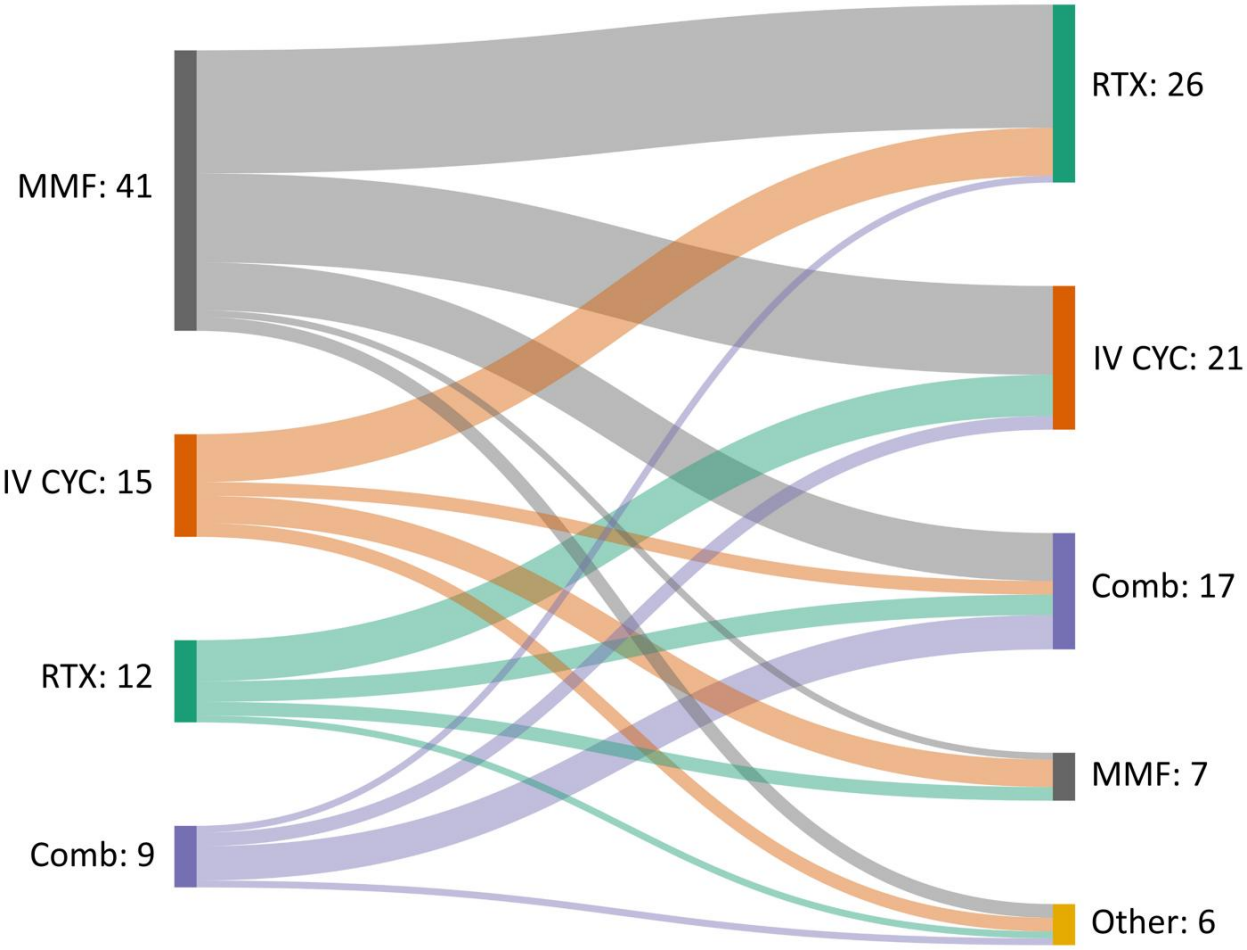
Sankey diagram showing respondents’ choices of immunosuppressive drugs as first-line and second-line treatment in class IV pre-menopause patients. First-line therapy: combination therapy: − MMF + RTX or i.v. CYC/-i.v. CYC + RTX or belimumab. Second-line therapy: combination therapy: − MMF + RTX, i.v. CYC or CNIs/− i.v. CYC + RTX + belimumab, MMF or AZA/− RTX + CNIs + AZA/− RTX + belimumab. Others: CNIs, AZA, oral CYC and belimumab. Comb: combination therapy

For pre-menopausal patients with renal impairment and a high activity index on biopsy, the most common first-line therapy was MMF [41 (53%)], with 20 (49%) opting for a target dose of 2 g/day and 21 (51%) for 3 g/day. The next preferred choices were i.v. CYC [15 (19.5%)] and RTX [12 (15.5%)]. All respondents who preferred CYC selected the EuroLupus regimen [17]. The most common second-line therapy for non-respondent patients was RTX [26 (33.7%)], followed by i.v. CYC [21 (27%)] or combination therapy [17 (22%)]. Of the 21 who selected CYC, 17 (81%) preferred the EuroLupus regimen and 4 (19%) the higher-dose National Institutes of Health (NIH) regimen [17]. Combination therapy was preferred by 9 (11.7%) respondents for first-line treatment and 17 (22%) for second-line therapy for non-responders. No respondents used calcineurin inhibitors (CNIs), ciclosporin or tacrolimus as first-line therapy, either alone or in combination (Fig. 1).

There was a difference between the choice of first-line therapy for post-menopausal and pre-menopausal patients in this scenario (*P* < 0.001). For post-menopausal patients, the most common first-line therapy was i.v. CYC [39 (50.6%)], followed by MMF [28 (36.4%)], with 13 (46.5%) opting for a target dose of 2 g/day and 15 (53.5%) preferring 3 g/day, and combination therapy [7 (9.1%)]. Unlike in pre-menopausal women, of the 39 who selected CYC, 84.6% preferred EuroLupus, 10.3% the NIH regimen and 5.1% the CYCLOPS vasculitis regimen.

If there was no clinical response, most respondents [55 (71.5%)] changed to second-line therapy at 3–6 months, 14 (18%) at 6–12 months and 8 (10.5%) at <3 months. Only 23 (30%) of respondents reported repeating the renal biopsy before starting second-line therapy and 17 (74%) would change their choice of the second-line therapy if the repeated biopsy showed a high chronicity index; of these 12 (70.6%) chose MMF, 3 (17.6%) chose AZA and 2 (11.8%) no immunosuppression, whereas previously with high activity on renal biopsy, they had chosen i.v. CYC [6 (35.3%)], RTX [4 (23.5%)] or combination therapy [7 (41.2%)].

In patients with class IV LN but without renal impairment, 34 (44%) respondents chose a different first-line induction regimen in the presence of renal impairment. Their new choices were as follows: MMF [27 (79%)], combination therapy [3 (9%)], AZA [2 (6%)], belimumab [1 (3%)] or no immunosuppressive therapy [1 (3%)] {these respondents had previously chosen i.v. CYC [11 (32.4%)], MMF [10 (29.4%)], RTX [9 (26.5%)] and combination therapy [4 (11.7%)] for class IV patients with renal impairment}.

For maintenance therapy, 65 (84.5%) respondents preferred MMF [with 50 (77%) opting for a target dose of 2 g/day and 15 (23%) for 3 g/day], 7 (9%) reported the use of AZA and 4 (6.2%) reported combination therapies. For more details and for differences between induction and maintenance therapy, see Supplementary Fig. S1, available at *Rheumatology Advances in Practice* online.

HCQ was used as part of the treatment regimen at induction by 50 (65%) respondents and at maintenance in 54 (70%). At induction, i.v. steroids alone were used by 19 (24.7%), oral steroids alone by 32 (41.6%) and both by 23 (29.9%) respondents. For maintenance therapy, 37 (48%) continued to use oral steroids, with the most frequent preference for an initial maintenance dose of 10 mg/day, reducing to 5 mg/day after 12 months.

### Treatment choices for class V LN

**Figure 2.**
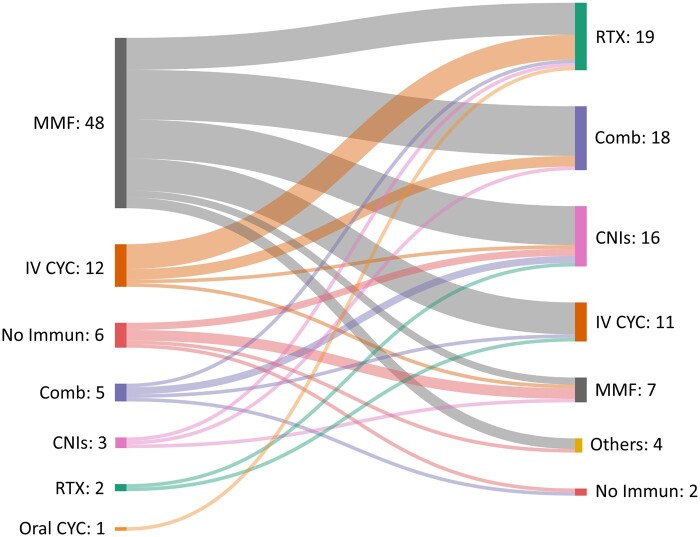
Sankey diagram showing respondents’ choices of immunosuppression as first-line and second-line treatment in class V nephrotic LN. First-line therapy: combination therapy: MMF + RTX or CNIs. Second-line therapy: Combination therapy: − MMF + RTX or CNIs/− i.v. CYC + RTX + CNIs or MMF/− RTX + CNIs + AZA/− CNIs + RTX. Others: AZA and oral CYC. Comb: combination therapy; No Immun: no immunosuppression

For patients with nephrotic-range proteinuria (>3 g/day), MMF [48 (62.3%)] was the preferred first-line choice [28 (58.4%) respondents using 2 g/day and 20 (41.6%) 3 g/day], followed by i.v. CYC 12 (15.6%) [11 (91.7%) preferred the Euro-Lupus regimen and 1 (8.3%) the NIH regimen]. A total of 5 (6.5%) respondents would not use immunosuppressive drugs. For non-responders, RTX [19 (24.7%)] followed by combination therapy [18 (23.4%)] and CNIs [16 (20.8%)] were the preferred second-line treatment (Fig. 2).

For maintenance therapy, 55 (71.4%) respondents reported the use of MMF [of these, 41 (74.5%) use 2 g/day and 14 (25.5%) use 3 g/day], 9 (11.6%) reported the use of combination therapy and 5 (6.5%) reported the use of CNIs only. For more details and for differences between induction and maintenance therapy, see Supplementary Fig. S2, available at *Rheumatology Advances in Practice* online.

HCQ was used as part of the treatment regimen at induction by 48 (62.6%) respondents and at maintenance by 46 (60%). At induction, i.v. steroids alone were used by 13 (16.9%), oral steroids alone by 47 (61%) and 9 (11.7%) respondents used both. In maintenance therapy, 32 (41.5%) continued to use oral steroids, with the most frequent preference being an initial maintenance dose of 10 mg/day and targeted dose of 5 mg/day after 12 months.

In patients with only modest proteinuria (<1 g/day), 44 (57%) respondents reported the use of MMF [of these, 26 (59%) use 2 g/day and 18 (41%) use 3 g/day], 16 (21%) reported that they do not use immunosuppressive drugs and 8 (10.4%) reported the use of combination therapy. For more details, see Supplementary Fig. S3, available at *Rheumatology Advances in Practice* online.

For maintenance therapy in this group, 46 (59.7%) selected MMF [36 (78%) of them use 2 g/day and 10 (22%) use 3 g/day], 16 (20.8%) reported that they do not use immunosuppressive drugs, 6 (7.8%) reported the use of combination therapy and 6 (7.8%) reported the use of AZA only. For more details and for differences between induction and maintenance therapy, see Supplementary Fig. S3, available at *Rheumatology Advances in Practice* online.

In non-nephrotic class V LN, HCQ was used as part of the induction regimen by 49 (63.6%) respondents and by 50 (65%) as part of maintenance therapy. At induction, i.v. steroids alone were used by 4 (5.2%), oral steroids alone by 46 (60%) and 2 (2.6%) respondents used both. For maintenance therapy, 22 (28.5%) continued to use oral steroids, with the most frequent preference being an initial maintenance dose of 10 mg/day and targeted dose of 5 mg/day after 12 months.

### Treatment preferences of nephrologists and rheumatologists

For services to support the treatment of LN, 20 (69%) nephrologists but only 13 (27%) rheumatologists reported having a formal departmental protocol for treating patients with LN (*P* < 0.001), 12 (41.4%) nephrologists and 25 (52%) rheumatologists reported having joint clinics for managing LN, 18 (62%) nephrologists and 36 (75%) rheumatologists reported having access to an MDT to discuss LN cases and 6 (20.7%) nephrologists and 20 (41.7%) rheumatologists reported having access to a specialized lupus nurse.

Renal biopsy would be arranged prior to starting the first treatment by 22 (76%) nephrologists and 36 (75%) rheumatologists and prior to changing to second-line therapy by 12 (41.4%) nephrologists and 11 (23%) rheumatologists.

The first-choice treatment of class IV LN in pre-menopausal female patients differed between nephrologists and rheumatologists; nephrologists preferred MMF (58.6%) followed by i.v. CYC (27.6%) then combination therapy (13.8%), while rheumatologists preferred MMF (50%) followed by RTX (25%) then i.v. CYC (14.6%) and combination therapy (10.4%) (*P* = 0.026) (Table 1).

**Table 1.** Comparison between nephrologists’ and rheumatologists’ choices regarding treatment of class IV LN patients

Characteristics	All respondents (*N* = 77)	Nephrologists (*n* = 29)	Rheumatologists (*n* = 48)	*P*-value
Usual first-line therapy for class IV LN in pre-menopausal females, *n* (%)				
MMF	41 (53.2)	17 (58.6)	24 (50)	**0.026**
i.v. CYC	15 (19.5)	8 (27.6)	7 (14.6)	
RTX	12 (15.6)	0 (0)	12 (25)	
Combination therapy: (MMF + RTX or i.v. CYC) and (i.v. CYC + RTX or belimumab)	9 (11.7)	4 (13.8)	5 (10.4)	
Time (in months) to change treatment if patients did not respond to first-line therapy, *n* (%)				
<3	8 (10.4)	3 (10.5)	5 (10.4)	**0.016**
3–6	55 (71.4)	16 (55)	39 (81.3)	
6–12	14 (18.2)	10 (34.5)	4 (8.3)	
Usual therapy for non-respondent class IV LN, *n* (%)				
MMF	8 (10.4)	3 (10.3)	5 (10.4)	0.50
i.v. CYC	21 (27.3)	8 (27.6)	13 (27)	
RTX	26 (33.8)	11 (37.9)	15 (31.3)	
Combination therapy (MMF + RTX, i.v. CYC or CNIs), (i.v. CYC + RTX + belimumab, MMF or AZA), (CNIs + AZA + RTX) and (RTX + belimumab)	15 (19.5)	3 (10.3)	12 (25)	
Others: CNIs, AZA, oral CYC and belimumab	7 (9)	4 (13.8)	3 (6.3)	
Usual maintenance therapy for class IV LN, n (%)				
MMF	65 (84.4)	24 (82.8)	41 (85.3)	
RTX	1 (1.4)	0 (0)	1 (2.1)	0.74
AZA	7 (9)	4 (13.8)	3 (6.3)	
Combination therapy: (AZA + MMF + CNIs) and (MMF + RTX, belimumab or AZA)	4 (5.2)	1 (3.4)	3 (6.3)	
Usual first-line therapy for class IV LN in post-menopausal females, *n* (%)				
MMF	28 (36.4)	13 (44.8)	15 (31.3)	
i.v. CYC	39 (50.6)	12 (41.4)	27 (56.3)	0.68
RTX	3 (4)	1 (3.4)	2 (4.2)	
Combination therapy (MMF + RTX or i.v. CYC), (i.v. CYC + RTX + MMF) and (belimumab + i.v. CYC or RTX)	7 (9)	3 (10.4)	4 (8.2)	

*P*-value is by chi-squared test with Monte Carlo simulation. Significant *P*-values (<0.05) are in bold.

For patients who failed to improve on first-line therapy, there was no difference between nephrologists’ and rheumatologists’ second-choice therapy; both preferred RTX (*P* = 0.50). However, rheumatologists were more likely to switch to second-line therapy earlier than nephrologists; 81.3% of rheumatologists chose to change after 3–6 months and 10.4% would change in <3 months, while 55% of nephrologists chose to change after 3–6 months and 34.5% would change after 6–12 months (*P* = 0.016).

Second-line therapy for refractory nephrotic-range class V LN differed between nephrologists and rheumatologists; nephrologists preferred CNIs alone, while rheumatologists preferred combination therapy (*P* = 0.041) (Table 2). There was no significant difference in HCQ use between rheumatologists and nephrologists (see Supplementary Table S1, available at *Rheumatology Advances in Practice* online). Comparisons between rheumatology consultants and trainees are presented in Supplementary Tables S2 and S3, available at *Rheumatology Advances in Practice* online.

**Table 2.** Comparison between nephrologists’ and rheumatologists’ choices regarding treatment of class V LN patients

Characteristics	All respondents (*N* = 77)	Nephrologists (*n* = 29)	Rheumatologists (*n* = 48)	*P*-value
Usual first-line therapy for class V LN with nephrotic syndrome, *n* (%)				
MMF	48 (62.3)	21 (72.4)	27 (56.3)	0.25
i.v. CYC	12 (15.6)	1 (3.4)	11 (22.8)	
Combination therapy: (MMF + RTX or CNIs)	5 (6.5)	2 (6.9)	3 (6.3)	
Others: oral CYC, CNIs and RTX	6 (7.8)	3 (10.3)	3 (6.3)	
No immunosuppressive drugs (only HCQ and/or steroids)	6 (7.8)	2 (6.9)	4 (8.3)	
Usual first-line therapy for class V LN without nephrotic syndrome, *n* (%)				
MMF	44 (57)	16 (55.2)	28 (58.3)	0.45
i.v. CYC	3 (4)	0 (0)	3 (6.3)	
Combination therapy: (MMF + CNIs, AZA or RTX) and (i.v. CYC + RTX)	8 (10.4)	2 (6.9)	6 (12.5)	
Others: RTX, CNIs and AZA	6 (7.8)	3 (10.3)	3 (6.3)	
No immunosuppressive drugs (only HCQ and/or steroids)	16 (20.8)	8 (27.6)	8 (16.6)	
Usual therapy for non-respondents class V LN with nephrotic syndrome, *n* (%)				
i.v. CYC	11 (14.3)	1 (3.4)	10 (20.8)	**0.041**
RTX	19 (24.7)	7 (24.1)	12 (25)	
CNI (tacrolimus or cyclosporine)	16 (20.8)	11 (37.9)	5 (10.4)	
Combination therapy: (MMF + RTX or CNIs), (i.v. CYC + RTX + CNIs or MMF), (RTX + CNIs + AZA) and (CNIs + RTX)	18 (23.3)	5 (17.2)	13 (27.1)	
Others: MMF, AZA and oral CYC	11 (14.3%)	4 (13.8%)	7 (14.6%)	
No immunosuppressive drugs (only HCQ and/or steroids)	2 (2.6%)	1 (3.4%)	1 (2.1%)	
Usual maintenance therapy for class V LN with nephrotic syndrome, *n* (%)				
MMF	55 (71.4)	19 (65.7)	36 (75)	
CNI (tacrolimus or cyclosporine)	5 (6.5)	3 (10.3)	2 (4.2)	
Combination therapy: (MMF + CNIs or AZA)	9 (11.6)	3 (10.3)	6 (12.4)	0.70
Others: RTX and AZA	5 (6.5)	3 (10.3)	2 (4.2)	
No immunosuppressive drugs (only HCQ and/or steroids)	3 (4)	1 (3.4)	2 (4.2)	
Usual maintenance therapy for class V LN without nephrotic syndrome, *n* (%)				
MMF	46 (59.8)	16 (55.2)	30 (62.5)	
CNI (tacrolimus or cyclosporine)	2 (2.6)	1 (3.4)	1 (2.1)	
Combination therapy: (MMF + CNIs or AZA)	6 (7.8)	0 (0)	6 (12.5)	0.20
Others: RTX and AZA	7 (9)	4 (13.8)	3 (6.3)	
No immunosuppressive drugs (only HCQ and/or steroids)	16 (20.8)	8 (27.6)	8 (16.6)	

*P*-value is by chi-squared test with Monte Carlo simulation. Significant *P*-values (<0.05) are in bold.

### Differences in treatment choices across the UK

The majority [72 (93.5%)] of the respondents were working in England and the remainder were working in Scotland [3 (3.9%)], Northern Ireland [1 (1.3%)] or Wales [1 (1.3%)] (Table 3). The only difference between regions of the UK was the availability of a joint clinic between nephrologists and rheumatologists for treating patients with LN; joint clinics were more likely to be conducted in London, the South West and Northern Ireland, while no joint clinics were reported by three respondents in the North East and one respondent in Wales (*P* = 0.01). There were no differences between regions regarding having formal departmental protocols for LN treatment (*P* = 0.15), having an MDT meeting to discuss LN treatment (*P* = 0.80) and having a specialized lupus nurse (*P* = 0.08) (Table 3). For respondents’ choices for treatment according to place of work, please see Supplementary Tables S4 and S5, available at *Rheumatology Advances in Practice* online.

**Table 3.** Access to specialized services for the management of LN between regions of the UK

Respondents’ workplace region of the UK	Respondents, *n* (%)	Having formal departmental protocol for LN treatment (yes), *n* (%)	Having joint clinics for managing LN (yes), *n* (%)	Having an MDT for discussing LN treatment (yes), *n* (%)	Having a specialized lupus nurse (yes), *n* (%)
Wales	1 (1.3)	1 (100)	0 (0)	0 (0)	0 (0)
Scotland	3 (3.9)	1 (33.3)	1 (33.3)	2 (66.7)	1 (33.3)
Northern Ireland	1 (1.3)	0 (0)	1 (100)	1 (100)	1 (100)
London	8 (10.4)	5 (62.5)	8 (100)	5 (62.5)	5 (62.5)
East Midlands	7 (9)	5 (71.4)	5 (71.4)	5 (71.4)	2 (28.6)
West Midlands	9 (11.7)	3 (33.3)	6 (66.7)	7 (77.8)	5 (55.6)
North East	3 (3.9)	0 (0)	0 (0)	3 (100)	2 (66.7)
North West	12 (15.6)	3 (25)	3 (25)	8 (66.7)	1 (8.3)
South East	13 (16.9)	8 (61.5)	4 (30.8)	7 (53.8)	2 (15.4)
South West	7 (9)	1 (14.3)	3 (42.9)	7 (100)	3 (42.9)
Wessex	1 (1.3)	0 (0)	1 (100)	1 (100)	1 (100)
Yorkshire and the Humber	12 (15.6)	6 (50)	5 (41.7)	10 (83.3)	3 (33)

## Discussion

Our survey results revealed that specialized services that aid in the treatment of LN patients, such as joint clinics and MDT meetings between nephrologists and rheumatologists, access to a dedicated lupus nurse or departmental protocols for the treatment of LN patients, are not available in all UK centres. We did not collect data about the size of individual centres and so a more comprehensive survey is needed to assess the landscape of services available to treat patients with LN. It is possible that physicians in some larger centres did not take part in the survey. That notwithstanding, any geographical variation needs to be addressed, either by establishing these services in hospitals that treat LN patients, or by establishing local or regional pathways for referral of those patients to the tertiary hospitals, as joint clinics and MDT meetings have been shown to reduce time to renal biopsy and improve the quality of acre [18].

About 25% of rheumatologists and 24% of nephrologists reported that they do not plan for renal biopsy before starting treatment of LN patients if the diagnosis appeared clear cut clinically. Understanding why a biopsy is not performed is important because guidelines recommend that a biopsy should be performed in all patients unless contraindicated. We believe that a renal biopsy should be considered for all patients with presumed LN prior to starting treatment, as renal involvement in SLE patients can also occur due to concomitant APS [19] or other glomerular diseases such as minimal change disease, IgA nephropathy, focal segmental glomerulosclerosis, thin basement membrane disease, amyloidosis or hypertensive nephropathy [20]. Biopsies are also important in determining prognosis and guiding management; e.g. a high chronicity index is associated with persistent proteinuria and reduced renal function that usually do not respond to immunosuppression [21].

Our survey results demonstrated that although most of the respondents follow established guidelines (BSR, EULAR/ERA-EDTA or KDIGO) [6–8], some physicians use other treatment regimens. The guidelines recommend induction treatment with MMF or i.v. CYC for class IV LN, however, 15.6% of respondents (all rheumatologists) reported the use of RTX alone and 11.6% of respondents (both rheumatologists and nephrologists) reported the use of combination therapy as first-line therapy in pre-menopausal patients. This may reflect that rheumatologists are more likely to manage patients with SLE who develop LN later in their disease course. These patients may have already received MMF and thus are eligible to receive RTX for new-onset LN according to both the current NHS England Clinical Commissioning Policy and BSR guidelines for the management of SLE [6]. However, we stated clearly in our case scenario that LN is the first presentation of lupus in the patient. We can’t exclude that some centres may have alternative funding pathways for RTX and/or the fear of CYC effects on fertility in pre-menopausal females may allow RTX to be used as first-line therapy, even though CYC with low doses (EuroLupus regimen) is unlikely to impact ovarian reserve [22]. For patients who do not respond to initial therapy, most of the respondents followed the guidelines that recommended a switch of the induction therapy between MMF and i.v. CYC or the use of RTX. It was also notable that many centres use 3 g/day as a target MMF dose both for induction and remission despite revision of induction guideline doses down to 2–3 g/day to improve safety and 2 g/day for maintenance.

For induction therapy in class IV post-menopausal females and for class IV maintenance therapy, respondents’ choices followed the guidelines. The most common choices for induction therapy in post-menopausal patients were i.v. CYC and MMF, and for maintenance the most common choice was MMF, except for a few respondents (6.5%) who chose RTX or combination therapy as maintenance therapy for patients after a good response to induction therapy. These choices may be due to their own treatment experience, or lack of formal departmental treatment protocols in their hospitals, as all these respondents reported having no departmental protocol for treating LN.

For class V LN, both the EULAR/ERA-EDTA and KDIGO guidelines suggest that MMF, i.v. CYC or CNIs can be used as alternatives for each other as first-line therapy for treating patients with nephrotic syndrome or proteinuria >1 g/day. This is with a particular endorsement for CNI, as it protects against podocyte injury as part of its antiproteinuric actions [23]. Our results showed that there was variation in respondents’ choices for treatment of those patients, with the highest percentage choosing MMF then i.v. CYC. Only a small number (7.8%) used CNIs alone as first-line treatment, but this increased to 21% as second-line therapy. The relatively low use of CNIs may be due to a lack data for these drugs in non-Asian populations [24, 25], fear of ciclosporin nephrotoxicity [26] or perhaps unfamiliarity with the drug.

Guidelines recommend the use of immunosuppressive drugs for class V patients if they have proteinuria >1 g/day. In our survey, only 21% of respondents (27.6% of nephrologists and 16.6% of rheumatologists) do not use immunosuppression for class V patients with proteinuria <1 g/day either in induction or maintenance. This may reflect concerns by physicians of disease progression or loss of renal function; however, studies in other types of glomerulonephritis have shown that for proteinuria to be a risk factor for renal progression, it is usually ≥1 g/day [27]. Reasons for using immunosuppressive drugs for these patients are the presence of non-renal disease that requires immunosuppression (although this was not a feature of our cases) or to assist in the reduction of steroid doses to avoid complications.

Despite the strong recommendation of the EULAR/ERA-EDTA guidelines for HCQ in the treatment of SLE patients, as it lowers the risk of flare [28], only 70% and 60–65% of respondents reported using it in maintenance therapy for class IV LN and class V LN, respectively. This may be due to concern for retinal toxicity with long-term HCQ use, especially in patients with renal impairment (eGFR <60 ml/min/1.73 m^2^) [29].

Although belimumab was approved for the treatment of LN in 2021 and is mentioned in both the updated 2019 EULAR/ERA-EDTA guidelines and KDIGO 2021 guidelines, very few respondents indicated a preference for belimumab in induction, maintenance or refractory disease. This may be due to unfamiliarity with belimumab and a preference for more established therapies.

It is important to note that the current national (BSR) guidelines were published in 2018 and refer to the 2012 EULAR/ERA-EDTA guidance. While the updated EULAR/ERA-EDTA 2019 and KDIGO 2021 guidelines are more recent, they predate the approval of belimumab and voclosporin for LN. Belimumab was approved for the treatment of LN by the European Commission in May 2021, while voclosporin was approved for use in the UK in November 2022, after our survey was concluded. Our results emphasize the need for more national discussion, agreement and common policies, including collection of outcomes-based data to help rheumatologists and nephrologists make evidence-based treatment decisions and reduce variation in clinical practice, as national and international guidelines continue to be updated.

Since our survey was conducted, the 2023 update of the EULAR guidelines has been published [30], which places greater emphasis on the role of belimumab than the 2019 guidelines. These updated guidelines also advocate more strongly for early combination therapy for LN. Overall, however, the message of the guidelines is that treatment decisions for LN should be made on an individual patient basis, considering access to treatment and patient preferences. Further studies will be needed to assess the impact of these guidelines on real-world management of LN.

In conclusion, there is variation in treatment choices for LN between physicians, with different choices reported by nephrologists and rheumatologists. These results suggest that clearer updated guidelines are required and the dissemination of these guidelines to all physicians who deal with LN patients is essential.

## Supplementary Material

rkae017_Supplementary_Data

## Data Availability

The raw data underlying this article will be made available upon reasonable request to the corresponding author.
