# Supplementary material for: Differences in management approaches for lupus nephritis within the UK
Source: Rheumatol Adv Pract. 2024 Feb 9;8(1):rkae017. doi: 10.1093/rap/rkae017 (PMC10926897; doi:10.1093/rap/rkae017)
Supplement: rkae017_Supplementary_Data [file rkae017_supplementary_data.docx]

**Supplementary Table S1:** Comparison between nephrologists’ and rheumatologists’ choices regarding the use of hydroxychloroquine (HCQ)

| Combine HCQ with immunosuppressive medications in: | All  (n = 77) | Nephrologists  (n = 29) | Rheumatologists  (n = 48) | P value |
| --- | --- | --- | --- | --- |
| Induction therapy for class Ⅳ LN | 50 (65%) | 22 (75.8%) | 28 (58.3%) | 0.12 |
| Maintenance therapy for class Ⅳ LN | 54 (70%) | 19 (65.5%) | 35 (73%) | 0.49 |
| Induction therapy for class Ⅴ LN | 48 (62.6%) | 17 (58.6%) | 31 (64.6%) | 0.60 |
| Maintenance therapy for class Ⅴ LN | 46 (60%) | 15 (51.7%) | 31 (64.6%) | 0.26 |

N number, LN lupus nephritis, HCQ hydroxychloroquine

P-value is by Chi Square test

**Supplementary Table S2:** Comparison between trainees’ and consultants’ choices regarding treatment of class Ⅳ LN patients

|  | All respondents (n = 77) | Trainees  (n = 10) | Consultants  (n = 67 ) | P value |
| --- | --- | --- | --- | --- |
| **Usual first-line therapy for class IV LN in pre-menopausal females, n (%)** |  |  |  |  |
| MMF | 41 (53.2%) | 4 (40%) | 37 (55%) |  |
| IV Cyclophosphamide | 15 (19.5%) | 1 (10%) | 14 (21%) | 0.15 |
| Rituximab | 12 (15.6%) | 4 (40%) | 8 (12%) |  |
| Combination therapy: (MMF + rituximab or IV cyclophosphamide) and (IV cyclophosphamide + rituximab or belimumab). | 9 (11.7%) | 1 (10%) | 8 (12%) |  |
| **Time to change treatment if patients did not respond to 1^st^ line therapy, N (%)** |  |  |  |  |
| < 3 months | 8 (10.4%) | 1 (10%) | 7 (10.4%) |  |
| 3-6 months | 55 (71.4%) | 7 (70%) | 48 (71.6%) | 0.98 |
| 6-12 months | 14 (18.2%) | 2 (20%) | 12 (18%) |  |
| **Usual therapy for non-respondents class IV LN, n (%)** |  |  |  |  |
| MMF | 8 (10.4%) | 2 (20%) | 6 (9%) |  |
| IV Cyclophosphamide | 21 (27.3%) | 6 (60%) | 15 (22.3%) |  |
| Rituximab | 26 (33.8%) | 1 (10%) | 25 (37.4%) |  |
| Combination therapy (MMF + rituximab, IV cyclophosphamide or CNIs), (IV cyclophosphamide + rituximab + belimumab. MMF or azathioprine), (CNIs + azathioprine + rituximab) and (rituximab + belimumab) | 15 (19.5%) | 0 (0%) | 15 (22.3%) | 0.11 |
| Others: CNIs, azathioprine, oral cyclophosphamide and belimumab | 7 (9%) | 1 (10%) | 6 (9%) |  |
| **Usual maintenance therapy for class IV LN, n (%)** |  |  |  |  |
| MMF | 65 (84.4%) | 9 (90%) | 56 (83.6%) |  |
| Rituximab | 1 (1.4%) | 0 (0%) | 1 (1.5%) | 0.56 |
| Azathioprine | 7 (9%) | 1 (10%) | 6 (9%) |  |
| Combination therapy: (azathioprine + MMF + CNIs) and (MMF + rituximab, belimumab or azathioprine) | 4 (5.2%) | 0 (0%) | 4 (5.9%) |  |
| **Usual first-line therapy for class IV LN in post-menopausal females, n (%)** |  |  |  |  |
| MMF | 28 (36.4%) | 1 (10%) | 27 (40.3%) |  |
| IV Cyclophosphamide | 39 (50.6%) | 7 (70%) | 32 (47.7%) | **0.02** |
| Rituximab | 3 (4%) | 2 (20%) | 1 (1.5%) |  |
| Combination therapy (MMF + rituximab or IV cyclophosphamide), (IV cyclophosphamide + rituximab + MMF), and(belimumab + IV cyclophosphamide or rituximab) | 7 (9%) | 0 (0%) | 7 (10.5%) |  |

N number, LN lupus nephritis, MMF Mycophenolate mofetil, CNI calcineurin inhibitors, IV intravenous

P-value is by Chi Square test with Monte Carlo simulation

P-value is significant when <0.05 (it is in bold when significant)

**Supplementary Table S3:** Comparison between trainees’ and consultants’ choices regarding treatment of class Ⅴ LN patients

|  | All respondents (n = 77) | Trainees  (n = 10) | Consultants  (n = 67) | P value |
| --- | --- | --- | --- | --- |
| **Usual first-line therapy for class V LN with nephrotic syndrome, n (%)** |  |  |  |  |
| MMF | 48 (62.3%) | 4 (40%) | 44 (65.5%) |  |
| IV Cyclophosphamide | 12 (15.6%) | 2 (20%) | 10 (15%) |  |
| Combination therapy: (MMF + rituximab or CNIs) | 5 (6.5%) | 1 (10%) | 4 (6%) | 0.09 |
| Others: oral cyclophosphamide, CNIs and rituximab | 6 (7.8%) | 0 (0%) | 6 (9%) |  |
| No immunosuppressive drugs (only hydroxychloroquine and/or steroid) | 6 (7.8%) | 3 (30%) | 3 (4.5%) |  |
| **Usual first-line therapy for class V LN without nephrotic syndrome, n (%)** |  |  |  |  |
| MMF | 44 (57%) | 4 (40%) | 40 (59.5%) |  |
| IV Cyclophosphamide | 3 (4%) | 2 (20%) | 1 (1.5%) |  |
| Combination therapy: (MMF + CNIs, azathioprine or rituximab) and (IV cyclophosphamide + rituximab) | 8 (10.4%) | 1 (10%) | 7 (10.5%) | 0.08 |
| Others: rituximab, CNIs and azathioprine | 6 (7.8%) | 0 (0%) | 6 (9%) |  |
| No immunosuppressive drugs (only hydroxychloroquine and/or steroid) | 16 (20.8%) | 3 (30%) | 13 (19.5%) |  |
| **Usual therapy for non-respondents class V LN with nephrotic syndrome, n (%)** |  |  |  |  |
| Iv cyclophosphamide | 11 (14.3%) | 2 (20%) | 9 (13.4%) |  |
| Rituximab | 19 (24.7%) | 2 (20%) | 17 (25.2%) |  |
| CNI (tacrolimus or cyclosporine) | 16 (20.8%) | 3 (30%) | 13 (19.5%) |  |
| Combination therapy: (MMF + rituximab or CNIs), (IV cyclophosphamide + rituximab + CNIs or MMF), (Rituximab + CNIs + azathioprine) and (CNIs + rituximab) | 18 (23.3%) | 0 (0%) | 18 (27%) | 0.72 |
| Others: MMF, azathioprine and oral cyclophosphamide | 11 (14.3%) | 2 (20%) | 9 (13.4%) |  |
| No immunosuppressive drugs (only hydroxychloroquine and/or steroid) | 2 (2.6%) | 1 (10%) | 1 (1.5%) |  |
| **Usual maintenance therapy for class V LN with nephrotic syndrome, n (%)** |  |  |  |  |
| MMF | 55 (71.4%) | 5 (50%) | 50 (74.5%) |  |
| CNI (tacrolimus or cyclosporine) | 5 (6.5%) | 1 (10%) | 4 (6%) |  |
| Combination therapy: (MMF + CNIs or azathioprine) | 9 (11.6%) | 1 (10%) | 8 (12%) | 0.06 |
| Others: rituximab and azathioprine | 5 (6.5%) | 1 (10%) | 4 (6%) |  |
| No immunosuppressive drugs (only hydroxychloroquine and/or steroid) | 3 (4%) | 2 (20%) | 1 (1.5%) |  |
| **Usual maintenance therapy for class V LN without nephrotic syndrome, n (%)** |  |  |  |  |
| MMF | 46 (59.8%) | 4 (40%) | 42 (62.5%) |  |
| CNI (tacrolimus or cyclosporine) | 2 (2.6%) | 0 (0%) | 2 (3%) |  |
| Combination therapy: (MMF + CNIs or azathioprine) | 6 (7.8%) | 1 (10%) | 5 (7.5%) | 0.46 |
| Others: rituximab and azathioprine | 7 (9%) | 2 (20%) | 5 (7.5%) |  |
| No immunosuppressive drugs (only hydroxychloroquine and/or steroid) | 16 (20.8%) | 3 (30%) | 13 (19.5%) |  |

N number, LN lupus nephritis, MMF Mycophenolate mofetil, CNI calcineurin inhibitors, IV intravenous

P-value is by Chi Square test with Monte Carlo simulation

P-value is significant when <0.05 (it is in bold when significant)

**Supplementary Table S4:** Comparison of respondents’ choices for treatment of class Ⅳ LN in different regions of UK

| **Respondents’ work place regions of UK** | **Usual first-line therapy for class IV LN in pre-menopausal females, n (%)** | **Usual therapy for non-respondents class IV LN, n (%)** | **Usual maintenance therapy for class IV LN, n (%)** | **Usual first-line therapy for class IV LN in menopausal females, n (%)** |
| --- | --- | --- | --- | --- |
| **Wales** |  |  |  |  |
| MMF | 1 (100%) | 0 (0%) | 1 (100%) | 1 (100%) |
| IV Cyclophosphamide | 0 (0%) | 0 (0%) | 0 (0%) | 0 (0%) |
| Rituximab | 0 (0%) | 1 (100%) | 0 (0%) | 0 (0%) |
| Combination therapy | 0 (0%) | 0 (0%) | 0 (0%) | 0 (0%) |
| Others | 0 (0%) | 0 (0%) | 0 (0%) | 0 (0%) |
| **Scotland** |  |  |  |  |
| MMF | 2 (66.7%) | 0 (0%) | 2 (66.6%) | 2 (66.7%) |
| IV Cyclophosphamide | 0 (0%) | 1 (33.3%) | 0 (0%) | 0 (0%) |
| Rituximab | 0 (0%) | 1 (33.3%) | 0 (0%) | 0 (0%) |
| Combination therapy | 1 (33.3%) | 0 (0%) | 1 (33.3%) | 1 (33.3%) |
| Others | 0 (0%) | 1 (33.3%) | 0 (0%) | 0 (0%) |
| **Northern Ireland** |  |  |  |  |
| MMF | 1 (100%) | 0 (0%) | 1 (100%) | 1 (100%) |
| IV Cyclophosphamide | 0 (0%) | 0 (0%) | 0 (0%) | 0 (0%) |
| Rituximab | 0 (0%) | 1 (100%) | 0 (0%) | 0 (0%) |
| Combination therapy | 0 (0%) | 0 (0%) | 0 (0%) | 0 (0%) |
| Others | 0 (0%) | 0 (0%) | 0 (0%) | 0 (0%) |
| **London** |  |  |  |  |
| MMF | 5 (62.5%) | 1 (12.5%) | 7 (87.5%) | 5 (62.5%) |
| IV Cyclophosphamide | 2 (25%) | 1 (12.5%) | 0 (0%) | 2 (25%) |
| Rituximab | 0 (0%) | 4 (50%) | 0 (0%) | 0 (0%) |
| Combination therapy | 1 (12.5%) | 2 (25%) | 0 (0%) | 1 (12.5%) |
| Others | 0 (0%) | 0 (0%) | 1 (12.5%) | 0 (0%) |
| **East Midlands** |  |  |  |  |
| MMF | 3 (42.9%) | 2 (28.6%) | 4 (57.1%) | 2 (28.6%) |
| IV Cyclophosphamide | 3 (42.9%) | 3 (42.8%) | 0 (0%) | 4 (57.1%) |
| Rituximab | 1 (14.2%) | 2 (28.6%) | 0 (0%) | 1 (14.3%) |
| Combination therapy | 0 (0%) | 0 (0%) | 0 (0%) | 0 (0%) |
| Others | 0 (0%) | 0 (0%) | 3 (42.9%) | 0 (0%) |
| **West Midlands** |  |  |  |  |
| MMF | 5 (55.6%) | 1 (11.1%) | 8 (88.9%) | 4 (44.4%) |
| IV Cyclophosphamide | 1 (11.1%) | 1 (11.1%) | 0 (0%) | 5 (55.6%) |
| Rituximab | 2 (22.2%) | 4 (44.4%) | 0 (0%) | 0 (0%) |
| Combination therapy | 1 (11.1%) | 3 (33.3%) | 1 (11.1%) | 0 (0%) |
| Others | 0 (0%) | 0 (0%) | 0 (0%) | 0 (0%) |
| **North East** |  |  |  |  |
| MMF | 2 (66.7%) | 0 (0%) | 2 (66.6%) | 2 (66.7%) |
| IV Cyclophosphamide | 0 (0%) | 0 (0%) | 0 (0%) | 0 (0%) |
| Rituximab | 0 (0%) | 1 (33.3%) | 0 (0%) | 0 (0%) |
| Combination therapy | 1 (33.3%) | 2 (66.6%) | 1 (33.3%) | 1 (33.3%) |
| Others | 0 (0%) | 0 (0%) | 0 (0%) | 0 (0%) |
| **North West** |  |  |  |  |
| MMF | 6 (50%) | 3 (25%) | 10 (83.3%) | 2 (16.7%) |
| IV Cyclophosphamide | 3 (25%) | 4 (33.3%) | 0 (0%) | 8 (66.7%) |
| Rituximab | 1 (8.3%) | 1 (8.3%) | 1 (8.3%) | 0 (0%) |
| Combination therapy | 2 (16.7%) | 1 (8.3%) | 1 (8.3%) | 2 (16.7%) |
| Others | 0 (0%) | 3 (25%) | 0 (0%) | 0 (0%) |
| **South East** |  |  |  |  |
| MMF | 7 (53.8%) | 0 (0%) | 13 (100%) | 4 (30.8%) |
| IV Cyclophosphamide | 3 (23.1%) | 6 (46.2%) | 0 (0%) | 6 (46.2%) |
| Rituximab | 2 (15.4%) | 4 (30.8%) | 0 (0%) | 2 (15.4%) |
| Combination therapy | 1 (7.7%) | 2 (15.4%) | 0 (0%) | 1 (7.7%) |
| Others | 0 (0%) | 1 (7.7%) | 0 (0%) | 0 (0%) |
| **South West** |  |  |  |  |
| MMF | 1 (14.3%) | 0 (0%) | 6 (85.7%) | 0 (0%) |
| IV Cyclophosphamide | 3 (42.9%) | 2 (28.6%) | 0 (0%) | 7 (100%) |
| Rituximab | 2 (28.6%) | 4 (57.1%) | 0 (0%) | 0 (0%) |
| Combination therapy | 1 (14.3%) | 0 (0%) | 0 (0%) | 0 (0%) |
| Others | 0 (0%) | 1 (14.3%) | 1 (14.3%) | 0 (0%) |
| **Wessex** |  |  |  |  |
| MMF | 0 (0%) | 0 (0%) | 1 (100%) | 0 (0%) |
| IV Cyclophosphamide | 0 (0%) | 1 (100%) | 0 (0%) | 1 (100%) |
| Rituximab | 0 (0%) | 0 (0%) | 0 (0%) | 0 (0%) |
| Combination therapy | 1 (100%) | 0 (0%) | 0 (0%) | 0 (0%) |
| Others | 0 (0%) |  | 0 (0%) | 0 (0%) |
| **Yorkshire and the Humber** |  |  |  |  |
| MMF | 8 (66.7%) | 1 (8.3%) | 10 (83.3%) | 5 (41.7%) |
| IV Cyclophosphamide | 0 (0%) | 2 (16.6%) | 0 (0%) | 6 (50%) |
| Rituximab | 4 (33.3%) | 3 (25%) | 0 (0%) | 0 (0%) |
| Combination therapy | 0 (0%) | 5 (41.7%) | 0 (0%) | 1 (8.3%) |
| Others | 0 (0%) | 1 (8.3%) | 2 (16.7%) | 0 (0%) |
| **P- value** | 0.58 | 0.51 | 0.39 | 0.34 |

N number, LN lupus nephritis, MMF Mycophenolate mofetil, CNI calcineurin inhibitors, IV intravenous

P-value is by Chi Square test with Monte Carlo simulation

P-value is significant when <0.05

**Supplementary Table S5:** Comparison of respondents’ choices for treatment of class Ⅴ LN in different regions of UK

| **Respondents’ work place regions of UK** | **Usual first-line therapy for class Ⅴ LN nephrotic, n (%)** | **Usual therapy for non-respondents class Ⅴ LN nephrotic, n (%)** | **Usual maintenance therapy for class Ⅴ LN nephrotic, n (%)** | **Usual first-line therapy for class Ⅴ LN with mild proteinuria, n (%)** | **Usual maintenance therapy for class Ⅴ LN with mild proteinuria, n (%)** |
| --- | --- | --- | --- | --- | --- |
| **Wales** |  |  |  |  |  |
| MMF | 1 (100%) | NA | 1 (100%) | 1 (100%) | 1 (100%) |
| IV Cyclophosphamide | 0 (0%) | 0 (0%) | NA | 0 (0%) | NA |
| CNIs | NA | 0 (0%) | 0 (0%) | NA | 0 (0%) |
| Rituximab | NA | 1 (100%) | NA | NA | NA |
| Combination therapy | 0 (0%) | 0 (0%) | 0 (0%) | 0 (0%) | 0 (0%) |
| Others | 0 (0%) | 0 (0%) | 0 (0%) | 0 (0%) | 0 (0%) |
| No immunosuppression | 0 (0%) | 0 (0%) | 0 (0%) | 0 (0%) | 0 (0%) |
| **Scotland** |  |  |  |  |  |
| MMF | 2 (66.7%) | NA | 2 (66.7%) | 1 (33.3%) | 1 (100%) |
| IV Cyclophosphamide | 0 (0%) | 0 (0%) | NA | 0 (0%) | NA |
| CNIs | NA | 0 (0%) | 0 (0%) | NA | 0 (0%) |
| Rituximab | NA | 3 (100%) | NA | NA | NA |
| Combination therapy | 0 (0%) | 0 (0%) | 0 (0%) | 0 (0%) | 0 (0%) |
| Others | 1 (33.3%) | 0 (0%) | 1 (33.3%) | 0 (0%) | 0 (0%) |
| No immunosuppression | 0 (0%) | 0 (0%) | 0 (0%) | 2 (66.7%) | 0 (0%) |
| **Northern Ireland** |  |  |  |  |  |
| MMF | 1 (100%) | NA | 1 (100%) | 1 (100%) | 1 (100%) |
| IV Cyclophosphamide | 0 (0%) | 0 (0%) | NA | 0 (0%) | NA |
| CNIs | NA | 1 (100%) | 0 (0%) | NA | 0 (0%) |
| Rituximab | NA | 0 (0%) | NA | NA | NA |
| Combination therapy | 0 (0%) | 0 (0%) | 0 (0%) | 0 (0%) | 0 (0%) |
| Others | 0 (0%) | 0 (0%) | 0 (0%) | 0 (0%) | 0 (0%) |
| No immunosuppression | 0 (0%) | 0 (0%) | 0 (0%) | 0 (0%) | 0 (0%) |
| **London** |  |  |  |  |  |
| MMF | 6 (75%) | NA | 8 (100%) | 4 (50%) | 6 (75%) |
| IV Cyclophosphamide | 0 (0%) | 0 (0%) | NA | 0 (0%) | NA |
| CNIs | NA | 3 (37.5%) | 0 (0%) | NA | 0 (0%) |
| Rituximab | NA | 3 (37.5%) | NA | NA | NA |
| Combination therapy | 2 (25%) | 2 (25%) | 0 (0%) | 2 (25%) | 0 (0%) |
| Others | 0 (0%) | 0 (0%) | 0 (0%) | 1 (12.5%) | 1 (12.5%) |
| No immunosuppression | 0 (0%) | 0 (0%) | 0 (0%) | 1 (12.5%) | 1 (12.5%) |
| **East Midlands** |  |  |  |  |  |
| MMF | 6 (85.7%) | NA | 5 (71.4%) | 4 (57.1%) | 3 (42.9%) |
| IV Cyclophosphamide | 0 (0%) | 1 (14.3%) | NA | 0 (0%) | NA |
| CNIs | NA | 2 (28.6%) | 1 (14.3%) | NA | 0 (0%) |
| Rituximab | NA | 1 (14.3%) | NA | NA | NA |
| Combination therapy | 0 (0%) | 1 (14.3%) | 0 (0%) | 0 (0%) | 0 (0%) |
| Others | 0 (0%) | 2 (28.6%) | 1 (14.3%) | 2 (28.6%) | 3 (42.9%) |
| No immunosuppression | 1 (14.3%) | 0 (0%) | 0 (0%) | 1 (14.3%) | 1 (14.3%) |
| **West Midlands** |  |  |  |  |  |
| MMF | 5 (55.6%) | NA | 7 (77.8%) | 6 (66.7%) | 7 (77.8%) |
| IV Cyclophosphamide | 3 (33.3) | 3 (33.3%) | NA | 1 (11.1%) | NA |
| CNIs | NA | 0 (0%) | 0 (0%) | NA | 0 (0%) |
| Rituximab | NA | 3 (33.3%) | NA | NA | NA |
| Combination therapy | 0 (0%) | 2 (22.2%) | 1 (11.1%) | 1 (11.1%) | 1 (11.1%) |
| Others | 1 (11.1%) | 1 (11.1%) | 1 (11.1%) | 0 (0%) | 0 (0%) |
| No immunosuppression | 0 (0%) | 0 (0%) | 0 (0%) | 1 (11.1%) | 1 (11.1%) |
| **North East** |  |  |  |  |  |
| MMF | 1 (33.3%) | NA | 2 (66.7%) | 2 (66.7%) | 2 (66.7%) |
| IV Cyclophosphamide | 0 (0%) | 0 (0%) | NA | 0 (0%) | NA |
| CNIs | NA | 0 (0%) | 1 (33.3%) | NA | 0 (0%) |
| Rituximab | NA | 1 (33.3%) | NA | NA | NA |
| Combination therapy | 0 (0%) | 1 (33.3%) | 0 (0%) | 0 (0%) | 1 (33.3%) |
| Others | 1 (33.3%) | 1 (33.3%) | 0 (0%) | 0 (0%) | 0 (0%) |
| No immunosuppression | 1 (33.3%) | 0 (0%) | 0 (0%) | 1 (33.3%) | 0 (0%) |
| **North West** |  |  |  |  |  |
| MMF | 7 (58.3%) | NA | 10 (83.3%) | 7 (58.3%) | 6 (50%) |
| IV Cyclophosphamide | 3 (25%) | 1 (8.3%) | NA | 0 (0%) | NA |
| CNIs | NA | 3 (25%) | 1 (8.3%) | NA | 1 (8.3%) |
| Rituximab | NA | 3 (25%) | NA | NA | NA |
| Combination therapy | 1 (8.3%) | 3 (25%) | 1 (8.3%) | 2 (16.7%) | 2 (16.7%) |
| Others | 1 (8.3%) | 1 (8.3%) | 0 (0%) | 1 (8.3%) | 0 (0%) |
| No immunosuppression |  | 1 (8.3%) | 0 (0%) | 2 (16.7%) | 3 (25%) |
| **South East** |  |  |  |  |  |
| MMF | 9 (69.2%) | NA | 8 (61.5%) | 5 (38.5%) | 6 (46.2%) |
| IV Cyclophosphamide | 0 (0%) | 2 (15.4%) | NA | 1 (7.7%) | NA |
| CNIs | NA | 1 (7.7%) | 0 (0%) | NA | 0 (0%) |
| Rituximab | NA | 1 (7.7%) | NA | NA | NA |
| Combination therapy | 1 (7.7%) | 3 (23.1%) | 3 (23.1%) | 2 (15.4%) | 1 (7.7%) |
| Others | 0 (0%) | 5 (38.5%) | 0 (0%) | 0 (0%) | 1 (7.7%) |
| No immunosuppression | 3 (23.1%) | 1 (7.7%) | 2 (15.4%) | 5 (38.5%) | 5 (38.5%) |
| **South West** |  |  |  |  |  |
| MMF | 3 (42.9%) | NA | 4 (57.1%) | 5 (71.4%) | 5 (71.4%) |
| IV Cyclophosphamide | 2 (28.6%) | 1 (14.3%) | NA | 0 (0%) | NA |
| CNIs | NA | 3 (42.9%) | 0 (0%) | NA | 0 (0%) |
| Rituximab | NA | 2 (28.6%) | NA | NA | NA |
| Combination therapy | 0 (0%) | 0 (0%) | 1 (14.3%) | 0 (0%) | 0 (0%) |
| Others | 1 (14.3%) | 1 (14.3%) | 1 (14.3%) | 0 (0%) | 0 (0%) |
| No immunosuppression | 1 (14.3%) | 0 (0%) | 1 (14.3%) | 2 (28.6%) | 2 (28.6%) |
| **Wessex** |  |  |  |  |  |
| MMF | 1 (100%) | NA | 1 (100%) | 1 (100%) | 1 (100%) |
| IV Cyclophosphamide | 0 (0%) | 0 (0%) | NA | 0 (0%) | NA |
| CNIs | NA | 0 (0%) | 0 (0%) | NA | 0 (0%) |
| Rituximab | NA | 1 (100%) | NA | NA | NA |
| Combination therapy | 0 (0%) | 0 (0%) | 0 (0%) | 0 (0%) | 0 (0%) |
| Others | 0 (0%) | 0 (0%) | 0 (0%) | 0 (0%) | 0 (0%) |
| No immunosuppression | 0 (0%) | 0 (0%) | 0 (0%) | 0 (0%) | 0 (0%) |
| **Yorkshire and the Humber** |  |  |  |  |  |
| MMF | 6 (50%) | NA | 6 (50%) | 7 (58.3%) | 7 (58.3%) |
| IV Cyclophosphamide | 4 (33.3%) | 3 (25%) | NA | 1 (8.3%) | NA |
| CNIs | NA | 0 (0%) | 2 (16.7%) | NA | 1 (8.3%) |
| Rituximab | NA | 3 (25%) | NA | NA | NA |
| Combination therapy | 1 (8.3%) | 6 (50%) | 3 (25%) | 1 (8.3%) | 1 (8.3%) |
| Others | 1 (8.3%) | 0 (0%) | 1 98.3%) | 2 (16.7%) | 2 (16.7%) |
| No immunosuppression | 0 (0%) | 0 (0%) | 0 (0%) | 1 (8.3%) | 1 (8.3%) |
| **P- value** | 0.65 | 0.32 | 0.79 | 0.97 | 0.79 |

N number, LN lupus nephritis, MMF Mycophenolate mofetil, CNI calcineurin inhibitors, IV intravenous, NA not applicable means that this choice is not present in the statistics alone but may be included in others option

P-value is by Chi Square test with Monte Carlo simulation

P-value is significant when <0.05


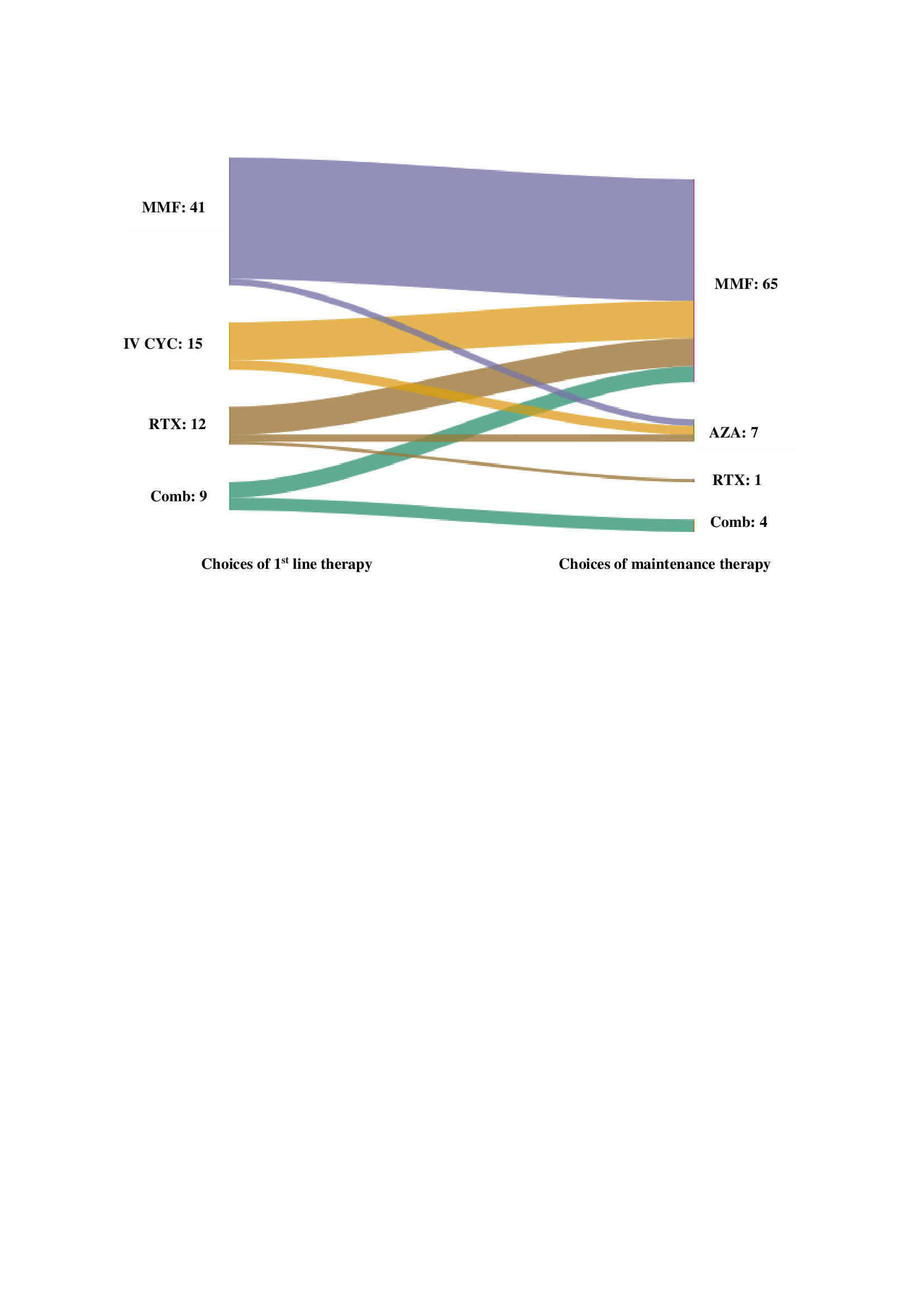


**Supplementary Figure S1:** A Sankey diagram that tracks respondents’ choices of immunosuppressive drugs as 1^st^ line and maintenance therapy for class Ⅳ LN.

1^st^ line therapy: Combination therapy: - MMF + RTX or IV CYC / -IV CYC + RTX or belimumab.

Maintenance therapy: Combination therapy: - MMF + RTX, AZA or belimumab / -MMF + AZA + CNIs

RTX Rituximab, Comb Combination therapy, MMF Mycophenolate Mofetil, AZA azathioprine, CNIs calcineurin inhibitors, IV CYC intravenous cyclophosphamide.


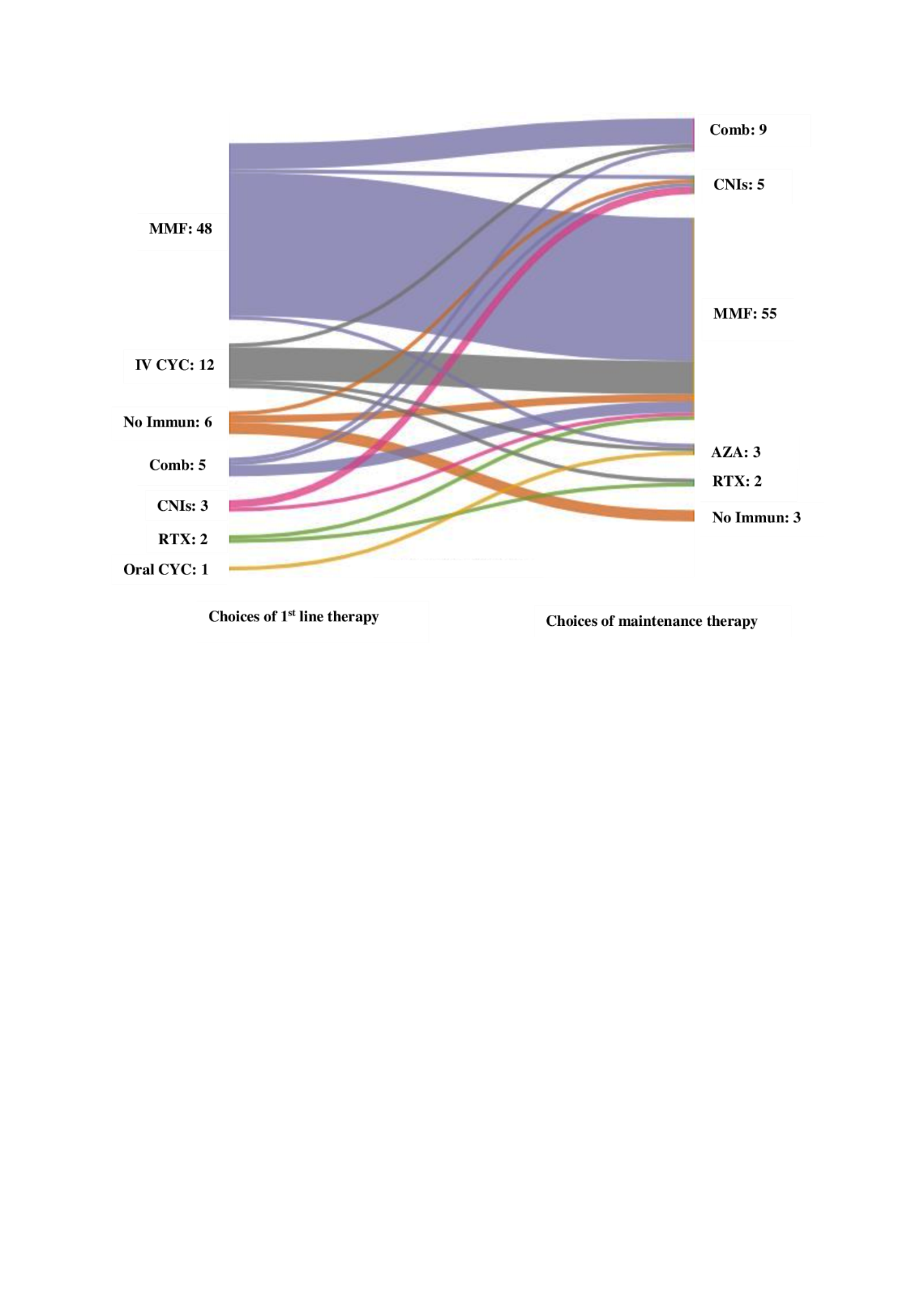


**Supplementary Figure S2:** A Sankey diagram that tracks respondents’ choices of immunosuppression as 1^st^ line and maintenance therapy for class Ⅴ nephrotic LN.

1^st^ line therapy: Combination therapy: MMF + RTX or CNIs

Maintenance therapy: Combination therapy: - MMF + CNIs or AZA

RTX Rituximab, Comb Combination therapy, MMF Mycophenolate Mofetil, CNIs Calcineurin Inhibitors, AZA azathioprine, IV CYC intravenous cyclophosphamide, Oral CYC oral cyclophosphamide, No Immun no immunosuppression.


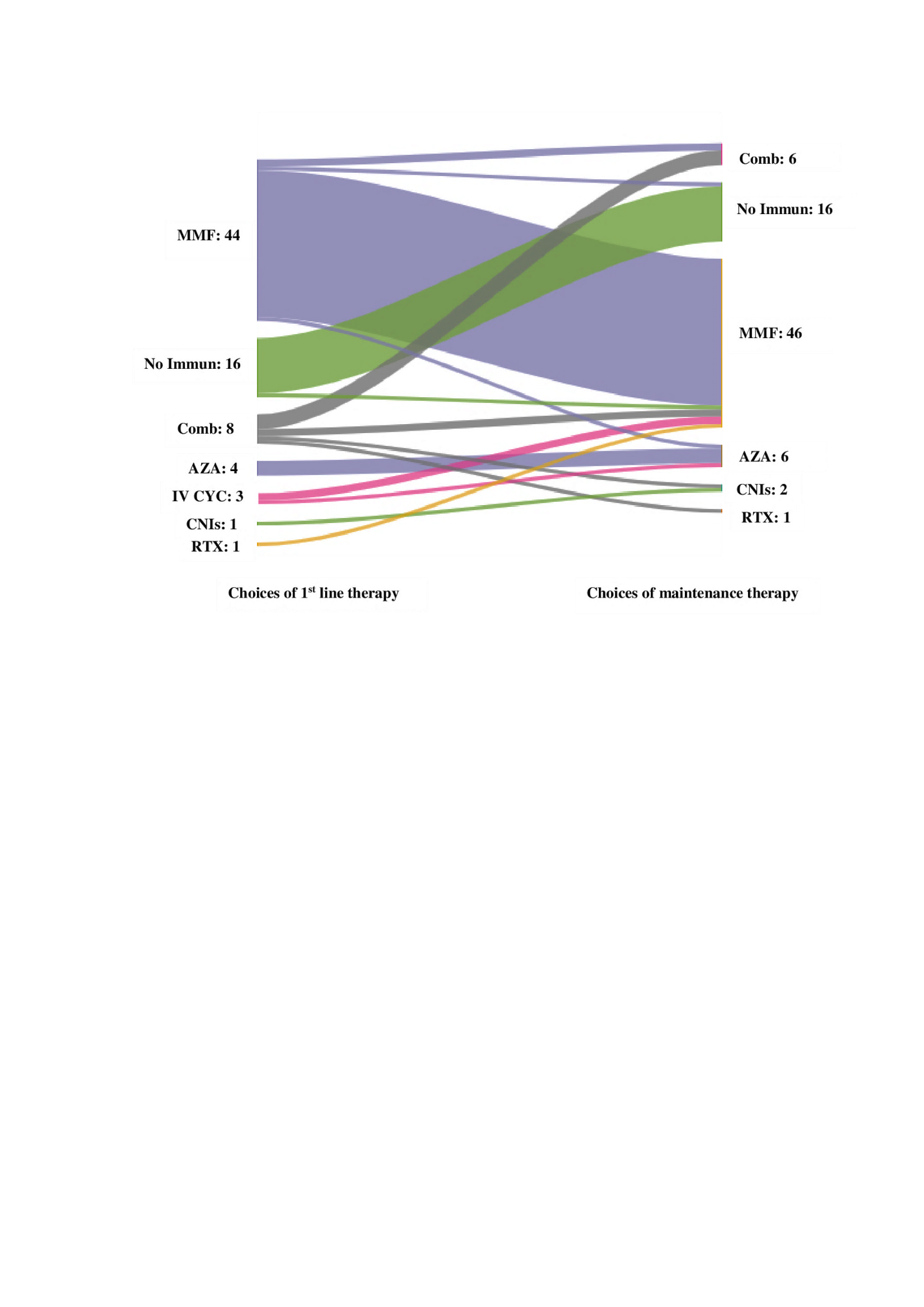


**Supplementary Figure S3:** A Sankey diagram that tracks respondents’ choices of immunosuppression as 1^st^ line and maintenance therapy for class Ⅴ LN with proteinuria < 1 g/day.

1^st^ line therapy: Combination therapy: -MMF + RTX, CNIs or AZA / - IV CYC + RTX

Maintenance therapy: Combination therapy: - MMF + CNIs or AZA

RTX Rituximab, Comb Combination therapy, MMF Mycophenolate Mofetil, CNIs Calcineurin Inhibitors, AZA azathioprine, IV CYC intravenous cyclophosphamide, No Immun no immunosuppression.

**Supplementary Data S1. Survey**

The survey

The aim of the questionnaire is to assess the practice of UK physicians who treat patients with lupus nephritis. So, please, answer the questions according to your usual daily practice not according to the best answer.

1- What is your specialty?

- Rheumatologist
- Nephrologist
- Others (please specify)…

2- What is your work level?

- Consultant
- Specialist (trainee)

3- Where do you work primarily?

- Wales
- Scotland
- Northern Ireland
- England

North East

North West

Yorkshire and the Humber

East Midlands

West Midlands

East of England

London

South East

South West

Wessex

4- Do you ever treat patients with lupus nephritis?

- Yes
- No

If yes

5- How many new lupus nephritis patients have you led treatment for over the past 12 months?

- ˂ 5
- 5- 10
- 11-20
- 21-30
- ˃30

6- Do you have a formal departmental protocol for managing lupus nephritis in your hospital?

- Yes
- No

7- Do you run joint clinics between nephrologists and rheumatologists for managing lupus nephritis in your hospital?

- Yes
- No

8- Do you have an MDT meeting where treatment plans for patients with lupus nephritis are discussed in your hospital?

- Yes
- No

9- Do you have specialized lupus nurses in your hospital?

- Yes
- No

10- In a patient with their first presentation of clear lupus nephritis (based on clinical and laboratory finding) would you always arrange a renal biopsy before planning treatment?

- Yes
- No
- CASE STUDY 1: A 24-years old female patient presented for first time with SLE and renal impairment. A renal biopsy showed class 4 lupus nephritis with high activity index (or you suspect class 4 from her clinical presentation). According to your usual practice in the management of lupus nephritis, please answer the following questions. Taking in consideration that she uses a reliable contraceptive method and does not plan to have children for the next 5 years:

11- What is your usual first treatment combination (induction regime) for this patient? (you can select more than one choice to build up a combination therapy)

- Hydroxychloroquine
- IV steroids (total dose up to 1g- 1.5g- 2.5g)
- Oral steroids ( initial dose of 0.3 mg/kg/day- 0.5mg/kg/day- 1mg/kg/day)
- IV cyclophosphamide (Euro-Lupus regimen- NIH regimen- other regimen)
- Oral cyclophosphamide
- Mycophenolate mofetil (target induction dose of 2g/day- 3g/day)
- Cyclosporin -Tacrolimus -Azathioprine
- Rituximab -Belimumab -Others (specify…)

12- If the patient responded well to the first treatment combination what is your usual maintenance therapy combination? (you can select more than one choice to build up a combination therapy)

- Hydroxychloroquine
- Oral steroids (please specify initial maintenance dose …..Target dose after 12 months…)
- Azathioprine - Oral cyclophosphamid
- Mycophenolate mofetil ( target maintenance dose of 2g/day – 3g/day)
- Cyclosporin - Tacrolimus - Rituximab
- Belimumab - Others (specify….)

13-If this patient failed to improve on the 1st treatment combination you have chosen, when would you decide to change to another combination of therapy? (Select your usual practice (in ≥ 50% of the cases))

- ˂3 months
- 3-6 months
- 6-12 months
- ˃12 months

14-What is your usual 2^nd^ choice treatment combination for this patient? (you can select more than one choice to build up a combination therapy)

- Hydroxychloroquine
- IV steroids (total dose up to 1g- 1.5g- 2.5g)
- Oral steroids ( initial dose of 0.3 mg/kg/day- 0.5mg/kg/day- 1mg/kg/day)
- IV cyclophosphamide (Euro-Lupus regimen- NIH regimen- other regimen)
- Oral cyclophosphamide
- Mycophenolate mofetil (target induction dose of 2g/day- 3g/day)
- Cyclosporin -Tacrolimus -Azathioprine
- Rituximab -Belimumab -Others (specify…)

15- Would you repeat the renal biopsy before starting the new treatment combination?

- No
- Yes

If yes

If the repeated renal biopsy for this patient showed that the chronicity index is high and the activity index is low, would you choose different treatment combination for this patient?

- No
- Yes

If yes

What is this combination?

- Hydroxychloroquine
- IV steroids (total dose up to 1g- 1.5g- 2.5g)
- Oral steroids ( initial dose of 0.3 mg/kg/day- 0.5mg/kg/day- 1mg/kg/day)
- IV cyclophosphamide (Euro-Lupus regimen- NIH regimen- other regimen)
- Oral cyclophosphamide
- Mycophenolate mofetil (target induction dose of 2g/day- 3g/day)
- Cyclosporin -Tacrolimus -Azathioprine
- Rituximab -Belimumab -Others (specify…)

16-If the same scenario was present in a menopausal female, what would be your usual first treatment combination (induction regime) for this patient? (you can select more than one choice to build up a combination therapy)

- Hydroxychloroquine
- IV steroids (total dose up to 1g- 1.5g- 2.5g)
- Oral steroids ( initial dose of 0.3 mg/kg/day- 0.5mg/kg/day- 1mg/kg/day)
- IV cyclophosphamide (Euro-Lupus regimen- NIH regimen- other regimen)
- Oral cyclophosphamide
- Mycophenolate mofetil (target induction dose of 2g/day- 3g/day)
- Cyclosporin -Tacrolimus -Azathioprine
- Rituximab -Belimumab -Others (specify…)

1. If the same scenario was present in an SLE patient without renal impairment (class 4 lupus nephritis with active urinary sediments, mild proteinuria and normal renal function) would you choose a different treatment combination?

- Yes
- No

If yes

What is this combination?

- Hydroxychloroquine
- IV steroids (total dose up to 1g- 1.5g- 2.5g)
- Oral steroids ( initial dose of 0.3 mg/kg/day- 0.5mg/kg/day- 1mg/kg/day)
- IV cyclophosphamide (Euro-Lupus regimen- NIH regimen- other regimen)
- Oral cyclophosphamide
- Mycophenolate mofetil (target induction dose of 2g/day- 3g/day)
- Cyclosporin -Tacrolimus -Azathioprine
- Rituximab -Belimumab -Others (specify…)
- CASE STUDY 2: A 50-years old female patient presented for the first time with SLE, mild renal impairment and heavy proteinuria ˃ 3 g/day. A renal biopsy showed class 5 lupus nephritis (or you suspect class 5 from her clinical presentation ) According to your usual practice please answer the following questions:

18- What is your usual first treatment combination (induction regime) for this patient? (you can select more than one choice to build up a combination therapy)

- Hydroxychloroquine
- IV steroids (total dose up to 1g- 1.5g- 2.5g)
- Oral steroids ( initial dose of 0.3 mg/kg/day- 0.5mg/kg/day- 1mg/kg/day)
- IV cyclophosphamide (Euro-Lupus regimen- NIH regimen- other regimen)
- Oral cyclophosphamide
- Mycophenolate mofetil (target induction dose of 2g/day- 3g/day)
- Cyclosporin -Tacrolimus -Azathioprine
- Rituximab -Belimumab -Others (specify…)

19- If the patient responded well to the first treatment combination what is your usual maintenance therapy combination? (you can select more than one choice to build up a combination therapy)

- Hydroxychloroquine
- Oral steroids (please specify initial maintenance dose …..Target dose after 12 months…)
- Azathioprine - Oral cyclophosphamide
- Mycophenolate mofetil ( target maintenance dose of 2g/day – 3g/day)
- Cyclosporin - Tacrolimus - Rituximab

Belimumab - Others ( specify….)

20- What is your usual 2^nd^ choice treatment combination if this patient did not respond well to the 1^st^ combination? (you can select more than one choice to build up a combination therapy)

- Hydroxychloroquine
- IV steroids (total dose up to 1g- 1.5g- 2.5g)
- Oral steroids ( initial dose of 0.3 mg/kg/day- 0.5mg/kg/day- 1mg/kg/day)
- IV cyclophosphamide (Euro-Lupus regimen- NIH regimen- other regimen)
- Oral cyclophosphamide
- Mycophenolate mofetil (target induction dose of 2g/day- 3g/day)
- Cyclosporin -Tacrolimus -Azathioprine
- Rituximab -Belimumab -Others (specify…)
- CASE STUDY 3: A 50-years old female patient presented for first time with SLE, mild renal impairment and mild proteinuria ˂ 1 g/day. A renal biopsy showed class 5 lupus nephritis. According to your usual practice answer the following questions:

21- What is your usual first treatment combination for this patient? (you can select more than one choice to build up a combination therapy)

- Hydroxychloroquine
- IV steroids (total dose up to 1g- 1.5g- 2.5g)
- Oral steroids ( initial dose of 0.3 mg/kg/day- 0.5mg/kg/day- 1mg/kg/day)
- IV cyclophosphamide (Euro-Lupus regimen- NIH regimen- other regimen)
- Oral cyclophosphamide
- Mycophenolate mofetil (target induction dose of 2g/day- 3g/day)
- Cyclosporin -Tacrolimus -Azathioprine
- Rituximab -Belimumab -Others (specify…)

22 - If patient responded well to the first treatment combination what is your usual maintenance therapy combination? (you can select more than one choice to build up a combination therapy)

- Hydroxychloroquine
- Oral steroids (please specify initial maintenance dose …..Target dose after 12 months…)
- Azathioprine - Oral cyclophosphamide
- Mycophenolate mofetil ( target maintenance dose of 2g/day – 3g/day)
- Cyclosporin - Tacrolimus - Rituximab

Belimumab - Others ( specify….)

23- Would you be happy to be contacted with a more in-depth questionnaire about treatment of patients with lupus nephritis?

- Yes
- No

If yes, please enter your email address below

Your email address will be stored separately from your answers on a secure server at the University of Birmingham for a period of up to 24 months after which it will be deleted. It will only be used to contact you about a further survey relating to lupus nephritis. If you change your mind and would like your email to be removed from the list, please contact:

1. Comments (optional)…………………………………………………….
